# Comparison of irritability, sleep and chronotype characteristics in children with Anxiety Disorder and ADHD

**DOI:** 10.1192/j.eurpsy.2023.362

**Published:** 2023-07-19

**Authors:** Ç. Yılmaz, S. Türkoğlu, Y. Gökçenoğlu

**Affiliations:** ^1^Çocuk ve ergen psikiyatrisi, selçuk üniversitesi, Konya; ^2^Psychiatry, Anamur State Hospital, Mersin, Türkiye

## Abstract

**Introduction:**

Attention deficit and hyperactivity disorder (ADHD) and Anxiety disorder (AD) are psychiatric conditions that should be kept in mind in every child and adolescent presenting with irritability. It is known that circadian rhythm disorders and especially the evening type chronotype are associated with ADHD and AD symptoms.

**Objectives:**

In this study, it was aimed to reveal the relationship between the chronotype and sleep habits and the level of irritability between the two groups in children and adolescents with AD and ADHD.

**Methods:**

In this cross-sectional study, 38 cases diagnosed with AD for the first time, 38 cases diagnosed with ADHD for the first time, and 76 healthy control groups without any psychiatric disorder or physical disease were included in this cross-sectional study. In the study, the sleep habits of the participants were evaluated with the Child Sleep Habits Questionnaire (CIAA); the chronotype preferences of the participants with the Child Chronotype Questionnaire (CCTQ) and the irritability levels of the children with the Affective Reactivity Index parent report form (ARI-P)

**Results:**

It was observed that the AD and ADHD groups had significantly higher ARI-P, CCTQ and CIAA scores compared to the control group. In the correlation analysis, when ADHD and AD were evaluated alone, no significant difference was found between CIAA and ARI-P. The ADHD group had higher CCTQ and ARI-P scores, although not statistically significant, compared to the AD group. Although there was no significant relationship in the AD group, a weak relationship was found between CIAA and ARI-P in the ADHD group.

**Conclusions:**

In our study, it was observed that the evening type chronotype was more prevalent in children with ADHD and AD, and sleep disorders and irritability were more common than the control group. When ADHD and AD groups were compared, no statistically significant difference was found. In the literature, it has been stated that evening chronotype carries a higher risk in terms of psychopathology, irritability seen at a young age can predict anxiety and mood disorders in adulthood, and irritability seen in ADHD can predict mood disorders that occur during follow-up. In this context, investigating the relationship between irritability, sleep disorders and chronotype on the basis of psychopathologies can make important contributions to the literatüre.

**Disclosure of Interest:**

None Declared

